# Supplementary figures and images for: Interferon lambda restricts herpes simplex virus skin disease by suppressing neutrophil-mediated pathology
Source: mBio. 2024 Mar 1;15(4):e02623-23. doi: 10.1128/mbio.02623-23 (PMC11005406; doi:10.1128/mbio.02623-23)

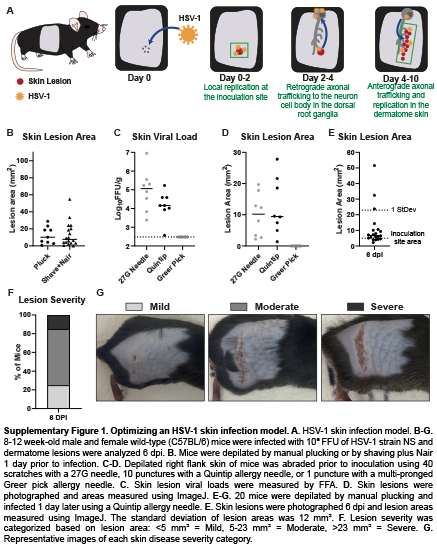

Supplement: Fig. S1 — Optimizing an HSV-1 skin infection model. [file mbio.02623-23-s0001.tif]

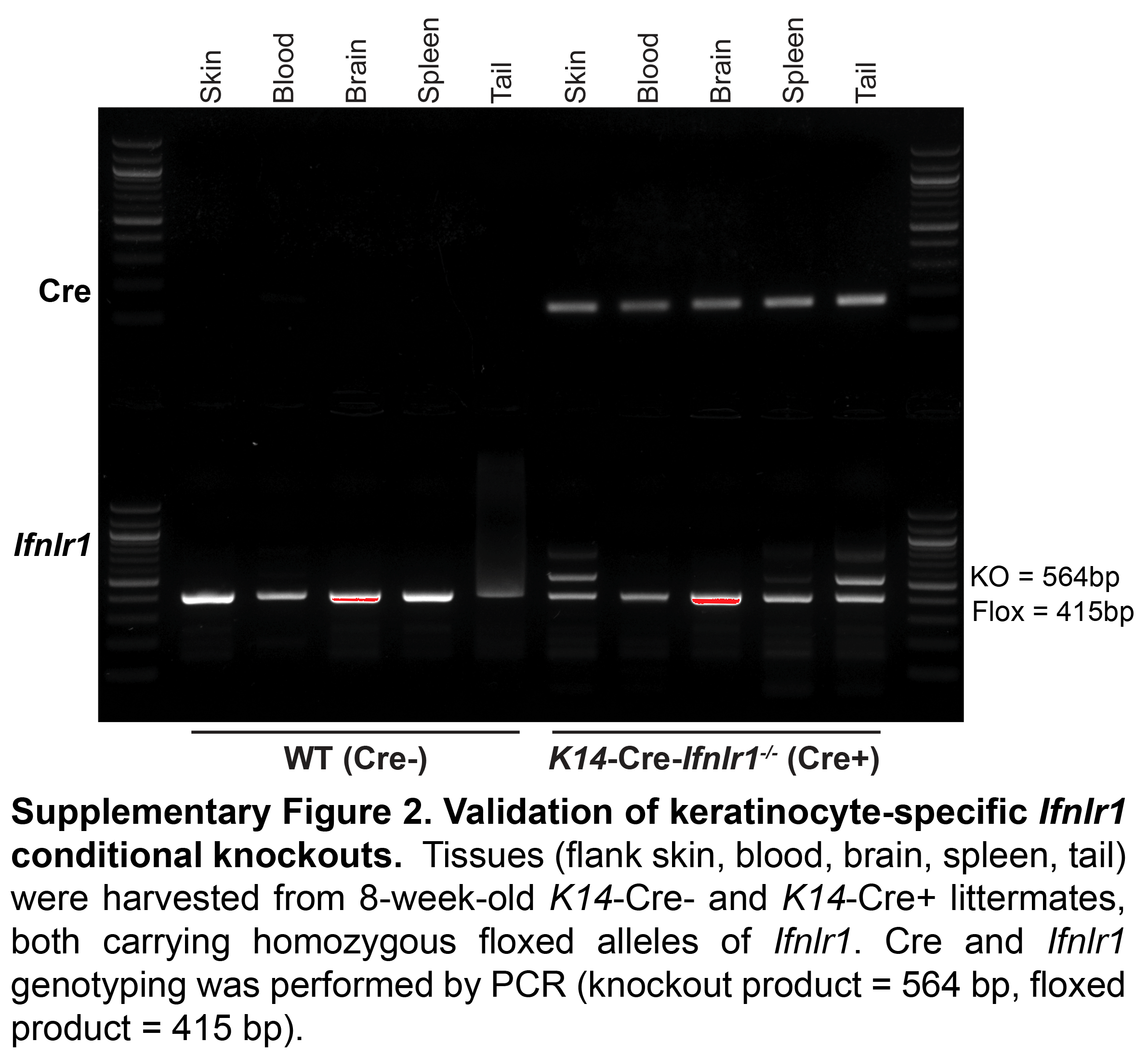

Supplement: Fig. S2 — Validation of keratinocyte-specific Ifnlr1 conditional knockouts. [file mbio.02623-23-s0002.tif]

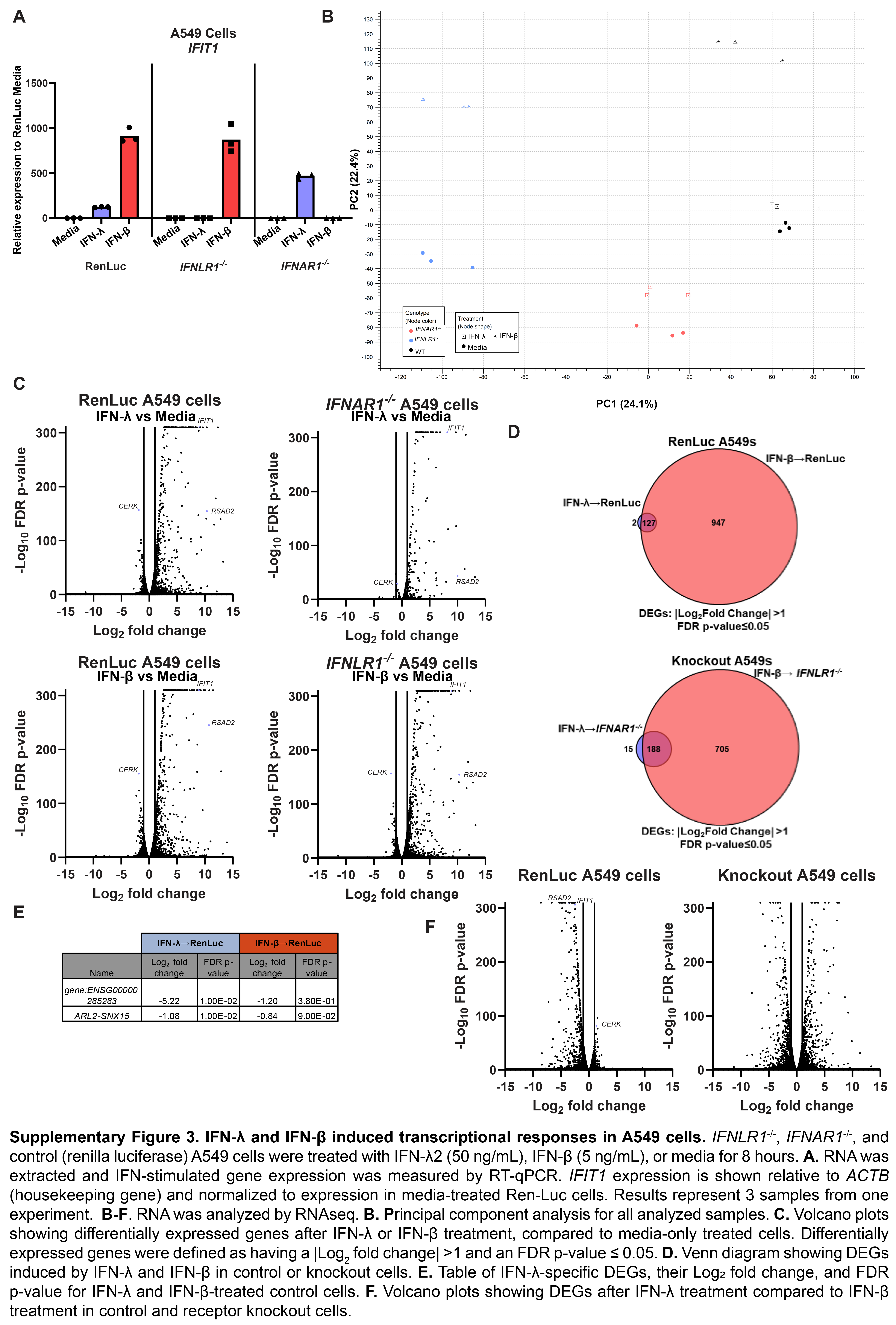

Supplement: Fig. S3 — IFN-λ and IFN-β induced transcriptional responses in A549 cells. [file mbio.02623-23-s0003.tif]

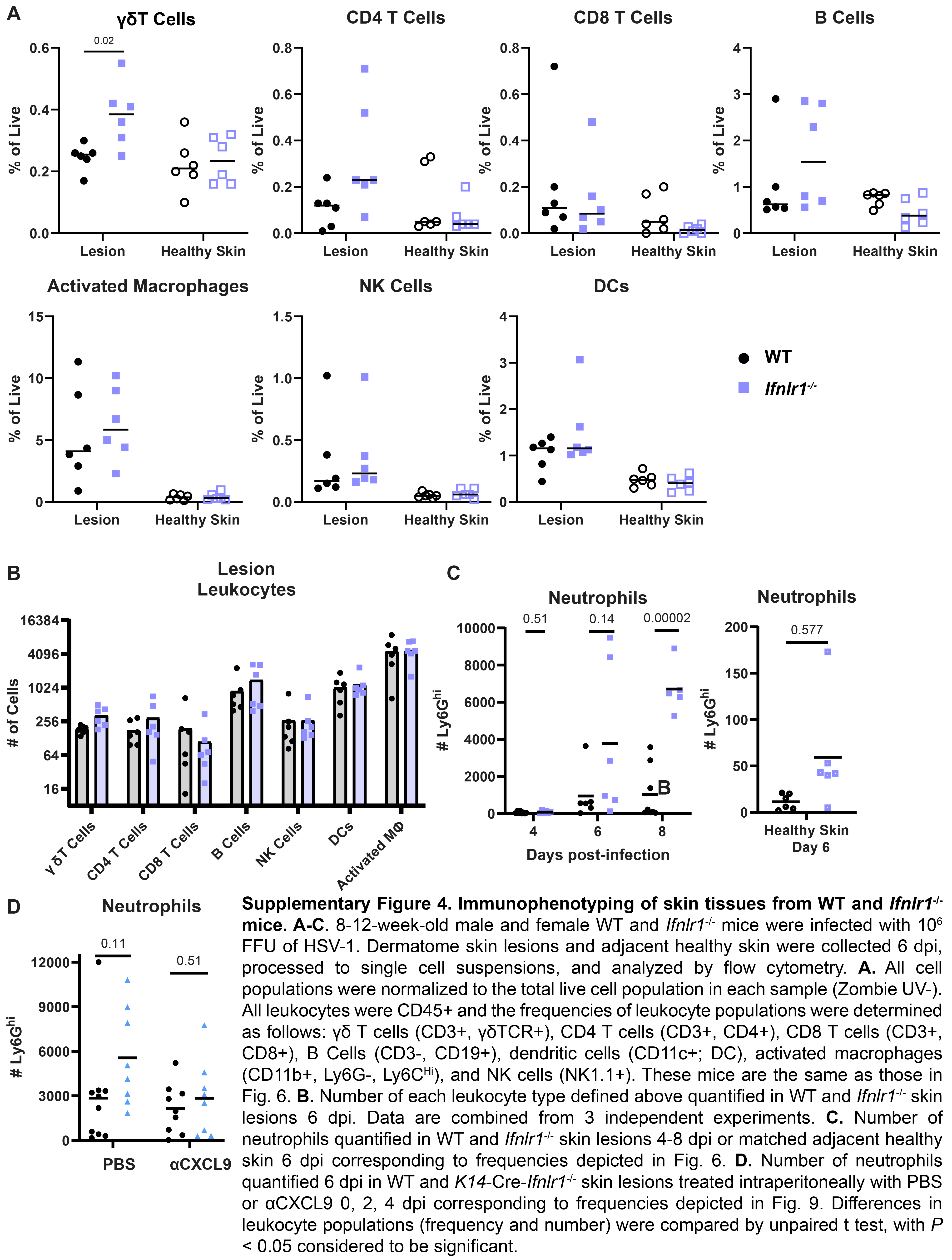

Supplement: Fig. S4 — Immunophenotyping of skin tissues from WT and Ifnlr1-/- mice. [file mbio.02623-23-s0004.tif]

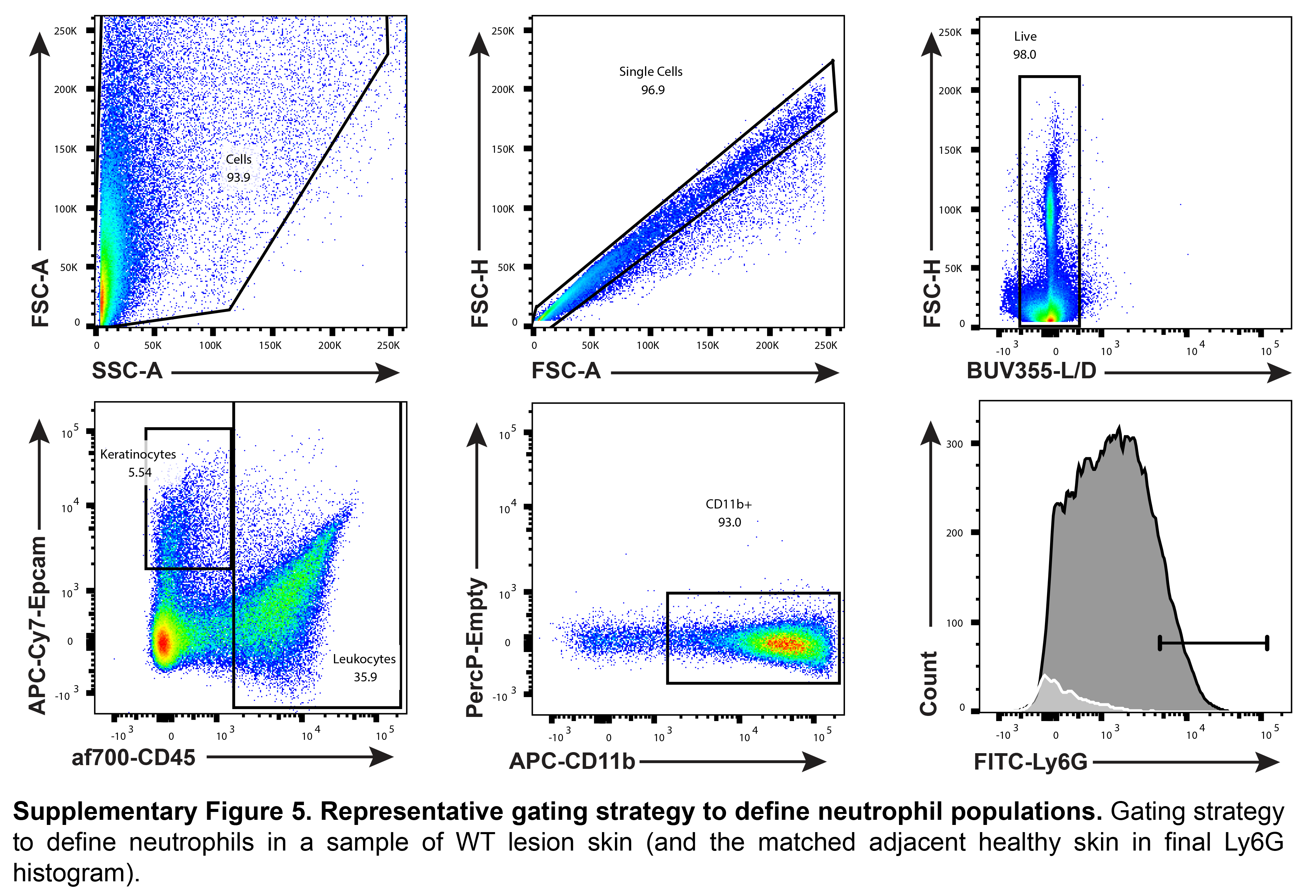

Supplement: Fig. S5 — Representative gating strategy to define neutrophil populations. [file mbio.02623-23-s0005.tif]
